# Screening of Pediatric Patients at Cardiology Clinic Identifies High Prevalence of Food Insecurity

**DOI:** 10.3390/ijerph23040437

**Published:** 2026-03-31

**Authors:** Alexander J. Kiener, Joseph Burns, Paul Cooper, Patrick Day, Derek Norton, Mounica Rao, Carlos Sanchez Parra, Thomas Seery, Keila Lopez, William B. Kyle, Shreya Sheth

**Affiliations:** 1 Division of Pediatric Cardiology, Department of Pediatrics, Baylor College of Medicine, Houston, TX 77030, USA; 2 Division of Pediatric Cardiology, Department of Pediatrics, University of Cincinnati College of Medicine, Cincinnati, OH 45229, USA; 3 Division of Pediatric Cardiology, Department of Pediatrics, Phoenix Children’s Hospital, Phoenix, AZ 85016, USA; 4 Division of Pediatric Cardiology, Department of Pediatrics, University of Colorado School of Medicine, Aurora, CO 80045, USA; 5 Division of Pediatric Cardiology, Department of Pediatrics, Warren Alpert Medical School of Brown University, Providence, RI 02903, USA

**Keywords:** food insecurity, congenital heart disease, social determinants of health

## Abstract

**Highlights:**

**Public health relevance—How does this work relate to a public health issue?**

- Food insecurity has negative implications for the health of children in the United States.
- Children with chronic medical conditions, including congenital heart disease, may have additional risk factors putting them at risk for food insecurity and/or risk of complications.

**Public health significance—Why is this work of significance to public health?**

- Food insecurity is noted to be highly prevalent via screening tools utilized at a US pediatric cardiology clinic.
- Food insecurity in a US pediatric cardiology clinic was significantly associated with social drivers of health, including Hispanic ethnicity, primary Spanish language, and limited neighborhood-level resource availability.

**Public health implications—What are the key implications or messages for practitioners, policy makers and/or researchers in public health?**

- The results of this study may inform food insecurity screening optimization and resource allocation in pediatric cardiology patients.
- Additional work should investigate for any impact of food insecurity on outcomes of patients with congenital heart disease.

**Abstract:**

Introduction: Food insecurity (FI) is a social driver that profoundly affects the health of children. Nutritional optimization is essential in patients with congenital heart disease (CHD). Material and Methods: We performed a cross-sectional survey screening for FI among patients aged 0–21 years at an outpatient pediatric cardiology clinic between September 2023 and December 2024. Sociodemographic and clinical data from encounters were collected, and diagnostic codes were used to classify CHD severity. The zip code-level median household income was determined using data from the U.S. Census. The Childhood Opportunity Index categorization was used to determine neighborhood-level resources. Univariate and multivariate logistic regression were used to assess sociodemographic associations with FI. Results: There were 955 encounters with completed FI screening. Positive screens were demonstrated in 200 surveys (20.9%). Compared to English-speaking White families, those with FI were more likely to be of Hispanic ethnicity (66% vs. 45.2%) and primarily speak Spanish (42.5% vs. 15.0%). Families with FI also lived in areas with lower median household income and fewer available resources. In multivariable analysis, after adjusting for ethnicity, income, and neighborhood-level resource availability, Spanish primary language was the only independent risk factor associated with FI (OR 2.7, 95% CI 1.7–4.2, *p* < 0.0001). There were no differences in FI status by CHD severity. Conclusions: FI was highly prevalent in this cohort and was associated with low-income and low-resource neighborhoods, Hispanic ethnicity, and a Spanish primary language. These results may have implications for targeting future FI interventions.

## 1. Introduction

Food insecurity (FI) describes the lack of access to adequate, nutritious, and affordable food [1]. FI is highly prevalent in the United States, affecting 17.9% of households with children under the age of 18 (6.5 million) in 2023 [2,3]. Numerous adverse health consequences of FI across the lifespan have been described. In children, FI is associated with worse self-rated health, impaired academic achievement, and increased risk of both hypertension and asthma [1,4,5]. In adulthood, FI has been associated with lower self-reported health and higher odds of adverse cardiovascular conditions, including coronary artery disease, angina, hypertension, and myocardial infarction [6].

Like many social drivers of health, the problem of FI is complex and influenced by several factors. While FI is strongly associated with household income, poverty is not the sole driver. In 2023, the United States Department of Agriculture (USDA) reported that 32.6% of households experiencing FI had household incomes at or above 185% of the poverty threshold [3]. It is also well-supported that single-parent families, Black and Hispanic families, families with young children (age < 6 years) and/or children with disabilities, and households in the Southern United States are at disproportionate risk for FI [3,7,8].

Food insecurity is especially relevant in the field of pediatric cardiology, as patients with congenital heart disease (CHD) are uniquely susceptible to the consequences of FI due to their increased metabolic demands, risk of growth impairment, and frequent need for specialized feeding plans [9,10]. Additionally, it is well-documented that children who are underweight or experiencing poor nutritional status are at significantly increased risk for morbidity when undergoing cardiac surgical interventions [9,10]. The financial burden associated with CHD-related healthcare costs has also been associated with FI [11].

The American Academy of Pediatrics (AAP) released a policy statement in 2015 (reaffirmed in 2021) to guide pediatric healthcare providers in efforts to mitigate the problem of FI [12]. In this statement, the AAP recommends that FI screening be performed at scheduled primary care health maintenance visits, or sooner if indicated. However, the burden of identifying and caring for children is the responsibility of all pediatric healthcare providers, not solely general pediatricians, as it is essential to recognize that FI carries specialty-specific implications.

However, the relationship between FI and outcomes in patients with CHD remains understudied. This study aimed to evaluate associations with FI among families with children who have CHD in a cohort of patients who were screened through a previously implemented FI screening initiative in a pediatric cardiology clinic.

## 2. Material and Methods

As a part of an FI quality improvement project, we conducted a cross-sectional study of patients aged 0–21 years who had an outpatient visit at the pediatric cardiology fellows’ clinic at Texas Children’s Hospital between September 2023 and December 2024. FI screening was attempted at every clinical encounter using the Hunger Vital Sign (HVS) tool, a validated 2-item questionnaire designed to assess for FI in the outpatient clinical setting [13,14]. Screening forms were available both in English and Spanish. A positive HVS screen was defined as any response other than “never” to one or more questions. Screening results were recorded in the electronic medical record.

All patients who screened positive for FI were provided a free online resource cultivated by Texas Children’s Hospital. This tool provides contact information for community resources in the patient’s zip code including food, employment, legal, housing, and financial resources, among others. A social work consultation for a more comprehensive needs assessment was left up to the clinic provider’s discretion. This study aims to describe the prevalence of FI in the community served and does not aim to evaluate the efficacy of this resource nor improvement in FI following provision of these interventions.

All clinical encounters with documented screening for FI were included in the study. Clinical data collected included ICD-10 diagnostic codes associated with the clinic visit. Diagnostic codes were used to determine the severity of cardiac diagnosis based on the categorization scheme proposed by Hoffman and associates [15]. Demographic data collected included sex, age, race, and ethnicity (as documented in the electronic medical record), primary language, insurance type, and home address zip code. Median household income was estimated based on household zip code using the 2020 US Census reports, which provide income data by residential area. Zip code-level median household income was then divided into quartiles. Household location was also used to identify the appropriate US census tract for each family screened and assign a corresponding overall Child Opportunity Index (COI), a composite grouping system evaluating neighborhood-level data across multiple domains relevant to children’s healthy development [16]. This data was indexed to metropolitan location groups by categories of “very low,” “low,” “moderate,” “high,” and “very high.”

Demographic and clinical data were compared between FI and non-FI groups using chi-square (or Fisher’s exact) and Wilcoxon rank sum testing. Univariable logistic regression was performed to identify associations with FI status. Variables meeting statistical significance on univariable analysis were included in multivariable logistic regression. Differences were considered statistically significant if they met a two-tailed *p* value < 0.05. Statistical analysis was performed using RStudio (Version 2023.12.0+369, Posit Software, PBC, Boston, MA, USA, 2023).

## 3. Results

A total of 955 out of 1745 clinical encounters documented completed screening of patients/families for FI (54.7%). Families with infants were less likely to have completed screening. However, patient demographics were otherwise similar between the two groups (Supplemental Table S1). Of those with completed screening, 200 patient encounters (20.9%) were positive for FI (Table 1).

There were no significant differences in age or sex based on FI status. The CHD severity was mild or none in most clinical encounters, without a difference between FI status. Families with FI were more likely to have public health insurance, self-pay, or another payment option when compared to those who were food secure (38.5% vs. 30.1%). Families with FI were also significantly more likely to be of Hispanic ethnicity and speak Spanish (66% and 42.5%, respectively) than non-FI families (45.2% and 15%, respectively). Zip code-level median household income was lower in FI families.

**Figure 1:**
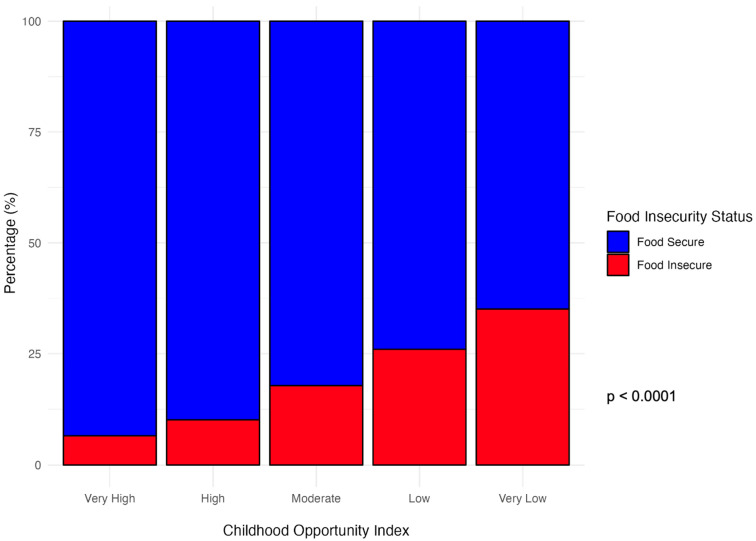
Food insecurity status by childhood opportunity index. Among patients screened for food insecurity status, percentages of patients from each Metropolitan level Childhood Opportunity Index are shown by food insecurity screening results.

The distribution of COI was associated with FI status as shown in Figure 1. Among patients living in “low” or “very low” COI neighborhoods, 31.8% reported FI compared to only 8% among patients in “high” or “very high” COI neighborhoods (*p* < 0.0001).

Univariate logistic regression results are shown in Table 2. FI was not associated with sex, young age, race, or CHD severity. Those with public insurance had 1.4 times higher odds of being food insecure than those with private insurance, which approached statistical significance (*p* = 0.06). Those who were self-payees had 4.3 times the odds of being food insecure compared to those with private insurance (*p* = 0.004). Families of Hispanic ethnicity and those that are primarily Spanish-speaking were associated with 2.5× increased odds of FI (*p* < 0.001) and 4.3× higher odds of FI (*p* < 0.001), compared to non-Hispanic families and English-speaking families respectively. There was an incremental increase in the odds of FI among decreasing quartiles of zip code-level median household income. FI status was inversely associated with increasing COI quartile, with an odds ratio of 0.4 (*p* = 0.03) among those of “very high” neighborhood resource status and an odds ratio of 2.7 (*p* < 0.001) among those of “very low” neighborhood resource status when compared to those in the “moderate” category.

Multivariable logistic regression was performed using variables significantly associated with univariable analysis, except for insurance status, due to low numbers and wide confidence intervals in several groups (Table 3). There was no significant association between FI and Hispanic ethnicity, median household income quartile, or COI. Spanish-speaking status remained significantly associated with FI after adjusting for additional factors (OR 2.7, *p* < 0.001).

## 4. Discussion

This study reported that among families screened for FI within a pediatric subspecialty clinic, over 20% screened positive for FI, which is significantly higher than the national average of 10% reported in recent studies [2]. This study did not find an association between CHD severity and the risk of FI. Importantly, however, our results show that social drivers, including Hispanic ethnicity, Spanish-speaking status, and living in areas with lower median household income and resource availability, demonstrated trends towards FI, with multivariate analysis revealing a statistically significant association with Spanish language and FI.

FI was significantly associated with Hispanic ethnicity and Spanish-speaking status (*p* < 0.0001). However, only Spanish-speaking status remained significantly associated with FI after adjusting for confounding factors in multivariable analysis. This suggests that language may serve as a structural barrier to accessing food resources, independent of ethnicity. Spanish-speaking families may face unique challenges, such as limited access to culturally appropriate and linguistically accessible support services, and fear or discomfort navigating systems that are not designed with their needs in mind, or potential impacts on legal status in the United States. For example, studies in accessibility to online health information showed decreased eHealth literacy in Spanish-speaking patients regarding colorectal cancer screening, and a lack of high-quality online Spanish language resources about sun safety and skin cancer [17,18]. A cross-sectional survey in pediatric cancer patients showed that Spanish language caregivers reported a higher frequency of changing or quitting jobs, higher delays in child education, and higher delays or avoidance of medical care due to concerns about the undocumented immigration status of household members than their English-speaking counterparts [19].

Importantly, CHD severity was not associated with FI, indicating that social risk does not necessarily correlate with medical complexity. Thus, universal screening for FI, rather than targeted screening based on medical severity, is likely to be the most sensitive screening methodology. It will be crucial for future studies to investigate the impact of FI on the clinical outcomes of children with CHD and to determine whether FI interventions can improve these outcomes.

We also found that families from lower-income quartiles and those living in areas with “very low” COI scores had higher unadjusted odds of FI. Although this was not significant in adjusted models, COI likely still serves as a proxy for systemic disadvantages. As the calculated *p*-value is at the cusp of statistical significance (*p* = 0.05), a better powered study may have revealed a significant association.

The COI is a particularly useful population-level metric for identifying high-need areas, and targeting these communities for proactive screening and resource allocation may help improve care equity. Prior studies have shown an association with COI and rehospitalization for common pediatric ambulatory conditions, and COI has been directly associated with a higher risk of FI in pediatric emergency centers [20,21]. Furthermore, children with the lowest COI scores have higher CHD in-hospital surgical mortality [16]. There may be a role in utilizing COI scores to risk-stratify these CHD patients concerning social needs, however more research is needed in this patient population.

Despite increased national attention to FI, gaps remain between screening and successful resource connection [1,7,22]. While referral rates may be high in some primary care settings, evidence suggests that families often do not receive, understand, or utilize the support offered to them. This may be particularly true in subspecialty clinics, where the perceived role of the provider may not traditionally include addressing social determinants.

Prior studies have reported the efficacy of FI referral for individuals and families with FI, showing higher community resource utilization, decreased future FI levels, as well as improvements in other social domains, such as employment, housing, and transportation [23,24,25,26]. Food banks are a commonly utilized referral and have been shown to play a role in mitigating the burdens of FI in the short term; however, they are often unable to meet the needs of families with chronic FI due to their limited resources and reliance on donations [27]. Other common referrals include federal programs, such as SNAP and WIC, which provide longer-term support to families but often have more stringent eligibility requirements [22,28]. Additional strategies include producing prescriptions, such as those available through the Gus Schumacher Nutrition Incentive Program, which provides these resources to individuals receiving support through SNAP or partnerships with farmers’ markets to promote access to nutritious food [28,29]. Importantly, providers and funding organizations have demonstrated an increasingly local focus on resource provision [30].

Universal screening for FI reveals a significant volume of affected families and, therefore, a significant burden on social workers and community programs. For this reason, engagement of key clinical and community partners is imperative to ensure that once identified, families are connected to appropriate community resources and to develop longitudinal relationships alongside active communication regarding the sustainability of these efforts.

### Limitations and Future Directions

There are several limitations of our study. First, this cross-sectional analysis was conducted only among patients who agreed to participate in screening. We only offered screening forms in English and Spanish, though our community demographics suggest a significant prevalence of Vietnamese and Arabic speakers, which may have contributed to exclusion bias. However, English and Spanish were the primary languages for 97% of patients seen in our clinic. In addition, as only 54.7% of encounters completed the screening, there is a component of sampling bias that may be present. However, there were minimal notable differences in patient demographics between those who were screened and those who were not screened. Second, this analysis included all clinical encounters from our cardiology clinic, including patients who were screened on multiple instances. We did not investigate the trends in FI screening results for these patients to see how the screening results changed over time. Further, we lack data regarding FI resource utilization, which is crucial in understanding how to optimize resource allocation efforts effectively. It is also important to note that our clinic is in a racially and ethnically diverse city in the southern United States. Therefore, while these findings are compelling, they may not be generalizable to other pediatric cardiology clinics nationwide.

Our study does not account for several variables that may be important modifiers of the observed trends. Many factors are known to affect CHD care and outcomes, including distance from the hospital, family structure, and parental education, which may also potentially impact FI [31,32]. Though not assessed in this study, future investigations should consider the inclusion of additional pertinent social drivers of health and a methodology to determine their interaction, as described.

Nevertheless, our study contributes to the body of evidence regarding the characteristics of patients and families with FI seen in a subspecialty pediatric clinic, which will help optimize screening efforts for FI.

## 5. Conclusions

FI is highly prevalent among families seen in pediatric cardiology clinic settings, particularly those who are Spanish-speaking, of Hispanic ethnicity, and from low-income or low-resource neighborhoods. There was no association between CHD severity and rates of FI. Our findings highlight the importance of integrating social determinants of health screening into routine cardiology and other subspecialty care, particularly for linguistically diverse populations. Further work is necessary to understand the impact of food insecurity on outcomes in children with CHD and to assess the impact of food insecurity interventions.

## Figures and Tables

**Table 1:** Patient demographics by food insecurity status.

	Food Secure (n = 755)	Food Insecure (n = 200)	*p*-Value
Insurance			0.009
Private	528 (69.9)	123 (61.5)	
Public	211 (27.9)	68 (34.0)	
Self-pay	8 (1.1)	8 (4.0)	
Other	8 (1.1)	1 (0.5)	
Sex			0.50
Female	347 (46.0)	86 (43.0)	
Infant	116 (15.4)	34 (17.0)	0.65
Age	5 (1, 12)	6.5 (1, 12.25)	0.21
Race			0.05
White	544 (72.1)	144 (72.0)	
Black or African American	153 (20.3)	41 (20.5)	
Asian	34 (4.5)	3 (1.5)	
Other	2 (0.3)	3 (1.5)	
Refused	3 (0.4)	0 (0)	
Unable to obtain	19 (2.5)	9 (4.5)	
Ethnicity			<0.0001
Hispanic or Latino	341 (45.2)	132 (66.0)	
Not Hispanic	397 (52.6)	61 (30.5)	
Refused	4 (0.5)	0 (0)	
Unable to obtain	13 (1.7)	7 (3.5)	
Language			<0.0001
English	624 (82.6)	110 (55.0)	
Spanish	113 (15.0)	85 (42.5)	
Other	14 (1.9)	4 (2.0)	
Unable to obtain	4 (0.5)	1 (0.5)	
Congenital Heart Disease Severity *			0.36
Mild or none	609 (80.7)	167 (83.5)	
Moderate to severe	146 (19.3)	33 (16.5)	
Median Household Income (×10^3^) †	69.7 (50.2, 91.2)	51.8 (43.5, 69.4)	<0.0001
Income Quartile †	[635]	[182]	<0.0001
Bottom	157 (24.7)	85 (46.7)	
Lower middle	142 (22.4)	42 (23.1)	
Upper middle	166 (26.1)	42 (23.1)	
Upper	170 (26.8)	13 (7.1)	

For categorical variable Chi-square test (or Fisher’s exact test if expected values < 5) to compare groups. For continuous variables Wilcoxon rank sum test was used. * Congenital heart disease severity based on Hoffman classification. † Median household income based on zip code and 2020 US Census data.

**Table 2:** Univariate logistic regression analysis of associations with food insecurity status.

	OR	95% CI	*p*-Value
Sex			
Female (ref)	-	-	-
Male	1.13	0.82–1.55	0.45
Infant	1.13	0.73–1.70	0.57
Insurance			
Private (ref)	-	-	-
Public	1.38	0.98–1.93	0.06
Self-Pay	4.29	1.55–11.88	0.004
Other	0.54	0.03–2.96	0.56
Congenital Heart Disease Severity *			
None (ref)	-	-	-
Mild	0.94	0.66–1.33	0.71
Moderate	0.85	0.50–1.40	0.53
Severe	0.72	0.35–1.38	0.35
Race			
White (ref)	-	-	-
Asian	0.33	0.08–0.94	0.07
Black or African American	1.01	0.68–1.49	0.95
Other/Not Available	1.54	0.66–3.33	0.28
Ethnicity			
Non-Hispanic (ref)	-	-	-
Hispanic or Latino	2.52	1.81–3.54	<0.0001
Other/Not Available	2.68	1.00–6.49	0.04
Language			
English (ref)	-	-	-
Spanish	4.28	3.02–6.04	<0.0001
Other	1.13	0.26–3.51	0.84
Income Quartile †			
Upper (ref)	-	-	-
Upper Middle	3.31	1.76–6.62	0.0004
Lower Middle	3.87	2.05–7.76	<0.0001
Bottom	7.08	3.92–13.76	<0.0001
Child Opportunity Index ‡			
Very High	0.39	0.16–0.86	0.03
High	0.47	0.20–1.06	0.08
Moderate (ref)	-	-	-
Low	1.49	0.83–2.74	0.19
Very Low	2.67	1.59–4.65	0.0003

Odds ratio = OR, Confidence interval = CI, reference = ref. * Congenital heart disease severity based on Hoffman classification. † Median household income based on zip code and 2020 US Census data. ‡ Child Opportunity Index normalized to metropolitan data.

**Table 3:** Multivariable logistic regression analysis of associations with food insecurity status.

	OR	95% CI	*p*-Value
Ethnicity			
Non-Hispanic (ref)	-	-	-
Hispanic or Latino	0.94	0.58–1.50	0.78
Other/Not Available	1.24	0.31–4.26	0.74
Language			
English (ref)	-	-	-
Spanish	2.66	1.70–4.20	<0.0001
Other	1.09	0.16–4.46	0.91
Income Quartile †			
Upper (ref)	-	-	-
Upper Middle	1.84	0.86–4.10	0.12
Lower Middle	1.48	0.63–3.57	0.37
Bottom	1.89	0.79–4.67	0.16
Child Opportunity Index ‡			
Very High	0.51	0.19–1.30	0.17
High	0.64	0.25–1.52	0.33
Moderate (ref)	-	-	-
Low	1.48	0.80–2.79	0.22
Very Low	1.90	1.00–3.71	0.05

Odds ratio = OR, Confidence interval = CI, reference = ref. † Median household income based on zip code and 2020 US Census data. ‡ Child Opportunity Index normalized to metropolitan data.

## Data Availability

Data may be made available on request to the authors (due to privacy concerns).

## Supplementary Materials

The following supporting information can be downloaded at: https://www.mdpi.com/article/10.3390/ijerph23040437/s1, Table S1: Patient Demographics by Food Insecurity Screening Status.
