# Supplementary material for: Screening of Pediatric Patients at Cardiology Clinic Identifies High Prevalence of Food Insecurity
Source: Int J Environ Res Public Health. 2026 Mar 31;23(4):437. doi: 10.3390/ijerph23040437 (PMC13115796; doi:10.3390/ijerph23040437)
Supplement: Supplementary file 1 [file ijerph-23-00437-s001.zip › ijerph-4138694-supplementary.pdf]

Supplementary Materials

Table S1. Patient Demographics by Food Insecurity Screening Status.

	Not Screened (n = 790)	Screened (n = 955)	p-value
**Insurance**			0.48
Private	534 (67.6)	651 (68.2)	
Public	225 (28.5)	279 (29.2)	
Self-pay	21 (2.7)	16 (1.7)	
Other	10 (1.3)	9 (0.9)	
**Sex**			0.23
Female	382 (48.4)	433 (45.3)	
**Infant**	175 (22.2)	150 (15.7)	**0.0007**
**Age**	5 (1, 12)	3 (1, 10)	**<0.0001**
**Race**			0.61
American Indian or Alaskan Native	3 (0.4)	1 (0.1)	
Asian	35 (4.4)	37 (3.9)	
Black or African American	142 (18.0)	194 (20.3)	
Native Hawaiian or Other Pacific Islander	1 (0.1)	0 (0)	
Other	5 (0.6)	4 (0.4)	
Refused	5 (0.6)	3 (0.3)	
Unable to obtain	24 (3.0)	28 (2.9)	
White	575 (72.8)	688 (72.0)	
**Ethnicity**			0.37
Hispanic or Latino	423 (53.5)	473 (49.5)	
Not Hispanic	346 (44.0)	458 (48.0)	
Refused	3 (0.4)	4 (0.4)	
Unable to obtain	18 (2.3)	20 (2.1)	
**Language**			0.78
Arabic	7 (0.9)	6 (0.6)	
Burmese	1 (0.1)	1 (0.1)	
Cantonese	0 (0)	1 (0.1)	
Dari	2 (0.3)	1 (0.1)	
English	595 (75.3)	734 (76.9)	
French	4 (0.5)	4 (0.4)	
French Creole	1 (0.1)	0 (0)	
Pashto	5 (0.6)	2 (0.2)	
Rohingya	1 (0.1)	0 (0)	
Somalian	0 (0)	1 (0.1)	
Spanish	171 (21.6)	198 (20.7)	
Tigrigna	0 (0)	2 (0.2)	
Unable to obtain	3 (0.4)	5 (0.5)	
**Congenital Heart Disease Severity** *			0.26
Mild or none	625 (79.1)	776 (81.3)	
Moderate to severe	165 (20.9)	179 (23.7)	

For categorical variable Chi-square test (or Fisher's exact if expected values < 5) to compare groups. For continuous variables Wilcoxon rank sum test was used.
* Congenital heart disease severity based on Hoffman classification.
